# Effect of Different Thawing Methods for Frozen Bull Semen and Additional Factors on the Conception Rate of Dairy Cows in Artificial Insemination

**DOI:** 10.3390/ani12182330

**Published:** 2022-09-07

**Authors:** Jacqueline Koch, Laura Patricia Weber, Maike Heppelmann, Fritjof Freise, Malte Klingelmann, Lisa Bachmann

**Affiliations:** 1 Alta Deutschland GmbH, 29525 Uelzen, Germany; 2 Clinic for Cattle, University of Veterinary Medicine Hannover, Foundation, 30559 Hannover, Germany; 3 Faculty of Agriculture and Food Science, University of Applied Science Neubrandenburg, 17033 Neubrandenburg, Germany; 4 Department for Biometry, Epidemiology and Information Processing, University of Veterinary Medicine Hannover, Foundation, 30559 Hannover, Germany; 5 Research-Institute for Farm Animal Biology (FBN), Institute of Nutritional Physiology “Oskar Kellner”, 18196 Dummerstorf, Germany

**Keywords:** artificial insemination, fertility, semen thawing

## Abstract

**Simple Summary:**

Today, recommendations for thawing methods for frozen bull semen vary, and clear data to assess their influence on fertility are contradictory. Under present practical conditions, no differences between the three different thawing methods could be detected, so no clear recommendation for semen thawing methods in dairy reproduction can be made based on our data. Study results provide evidence that inadequate reproductive performance of high-producing, lactating dairy cows is a multifactorial consequence of farm and fertility management; accordingly, the livestock industry should focus more on cow health to avoid reproductive disorders and further improve the reproductive performance of dairy cows in the future.

**Abstract:**

Recommendations for thawing methods of frozen bovine semen vary and clear data evaluating their influence on fertility are contradictory. In this respect, the aim of this study was to investigate the influence of different thawing methods of frozen bull semen in artificial insemination (AI) of dairy cows on conception rate (CR) under practical conditions and to determine further possible influencing factors on the success of AI in order to provide recommendations for practical use. From 2017 to 2019, 3393 AI were performed in a dairy farm in eastern Germany, distributed randomly into three groups of thawing methods: group A: *n* = 426 (11 s, 38 °C water bath); group B: *n* = 348 (35 s, 38 °C water bath); group C: *n* = 385 (30 s, “in the cow”). We observed no significant difference in CR from the general linear mixed model between the thawing methods (method A/B/C, 28.5%/26.6%/24.7%), but data analysis revealed effects of lactation number, month of insemination and AI method (natural heat vs. OvSynch) on CR. Based on our data, no clear recommendation for semen thawing method in dairy reproduction can be made. Our findings suggest that the main factors of influencing reproductive performance in the field are represented by the cow-side of fertility, e.g., insemination in natural heat, lactation number and season of insemination. Therefore, dairy farmers should focus more on cow conditions to further improve reproductive performance.

## 1. Introduction

Today, artificial insemination (**AI**) is the global standard in dairy livestock production. In the early 20th century, the development of AI revolutionized human and animal reproduction technology and had a tremendous impact worldwide [1]. The majority (95%) of AI is performed with frozen-thawed bull semen [2], which corresponds to about 130 million inseminations in cattle production per year [3]. The profitability of dairy farms is directly influenced by their reproductive efficiency [1].

The success of AI is multifactorial and depends on diverse factors of farm and fertility management. The spermatozoa count (studies could not detect further fertility improvement from 10 × 10^6^ spermatozoa [4,5]), the error rate in heat detection [6], a correct performed insemination by the technicians [7], subclinical endometritis [8,9], the body condition score, as well as peri- and postpartum health disorders influence the fertility of the inseminated cows [10,11,12].

Additionally, thawing technique of frozen semen for AI has a significant impact on the fertility of bull semen and thus, on the reproductive performance of dairy herds. Previous studies about the effect of different thawing methods on the fertility of bull semen included mostly laboratory experiments, only few studies contained field trials [13,14,15,16]. Given to the present processing, freezing and thawing methods of sperm dosages, only approximately 50% of the cryopreserved semen are recoverable after thawing [17,18]. In recent years, many in vitro studies showing that faster thawing at higher temperatures for shorter intervals resulted in better sperm quality and longer vitality of the thawed semen compared to slower thawing [19,20]. Under practical conditions, however, thawing at temperatures above 38 °C poses the risk of harmful heating and is therefore unsuitable. Meanwhile, mainly two different methods on how to thaw bull semen are established in practice. Remarkably, in the USA thawing is performed usually at 35–38 °C for >30 s [9,21], whereas in Germany the 0.25 mL straws are predominantly thawed at 37.5–38.5 °C for 10–12 s [22]. 

This development is based on two studies conducted by Berndtson et al. (1976) and Almquist et al. (1979) [13,15]. In 1976, Berndtson and colleagues demonstrated limited further improvement in the fertility of bull semen at thawing temperatures above 37 °C, whereupon they recommended a thawing temperature of 35–37 °C in order to protect the sperm from excessive harmful heating. In addition, they advised to avoid a semen temperature above 5 °C in the water bath to prevent cold shock from repeated cooling [15]. Few years later, Almquist and colleagues compared the fertility of frozen bull semen in 0.3 mL Continental-Straws thawed for either 12 or 30 s in a 32–35 °C water bath according to the 66-day nonreturn rate (**NRR**) of 24,424 first inseminations. In fact, they detected significantly better fertility of sperm thawed for 30 s (72%) by reaching a temperature of 32 °C, compared to semen thawed for 12 s (70.1%), related to a final temperature of 0 °C [13]. Further research with different straws confirmed those results [14,15,16]. According to the “cold shock” theory of Berndtson et al., in Germany, semen is thawed for 10–12 s. In the USA, thawing times of >30 s refer to the studies that determined better NRR after longer thawing intervals. 

Moreover, another thawing method described in the literature provides an attractive time-saving alternative to the previously described thawing procedures containing a water bath: thawing frozen bull semen “in the cow” [23]. In this technique, the semen portion is taken from the liquid nitrogen container and inseminated immediately; thus, the semen is thawed right in the genital tract of the cow. However, due to variations in environmental temperatures and time periods before insemination, the semen used in the “thaw-in-the-cow” method is affected by multiple variables. Nevertheless, a study with 23 heifers revealed no difference in fertility compared to thawing frozen bull semen in a water bath (34.4–36.7 °C, ≥30 s) [23]. In addition, another study likewise avoided thawing frozen semen in a water bath and instead used a thermally protected pocket to thaw the semen for 2–3 min. Among the 11,215 AI of heifers, no significant difference in the NRR was observed between frozen bull semen thawed with or without warm water [9].

Commercial breeding companies currently recommend different thawing methods for frozen semen in AI breeding (USA: 35–38 °C for >30 s; Germany: 37.5–38.5 °C for 10–12 s). As introduced previously, many studies meanwhile focused on different thawing techniques for frozen bull semen; however, only a few studies contained field trials and were limited in that they used NRR, an imprecise methodology, to assess fertility. To address these limitations, this study investigates three different thawing methods for AI with frozen bull semen, based on the conception rate (**CR**), to identify the technique providing the best fertility results. In addition, further animal- and management-related factors were analyzed to determine their influence on fertility in dairy farms. According to the results of previous studies in vitro and in vivo, we hypothesized that thawing semen for >30 s would result in higher conception rates in practice than thawing semen for a shorter time interval or “in the cow”.

## 2. Materials and Methods

### 2.1. Animals

**Figure 1:**
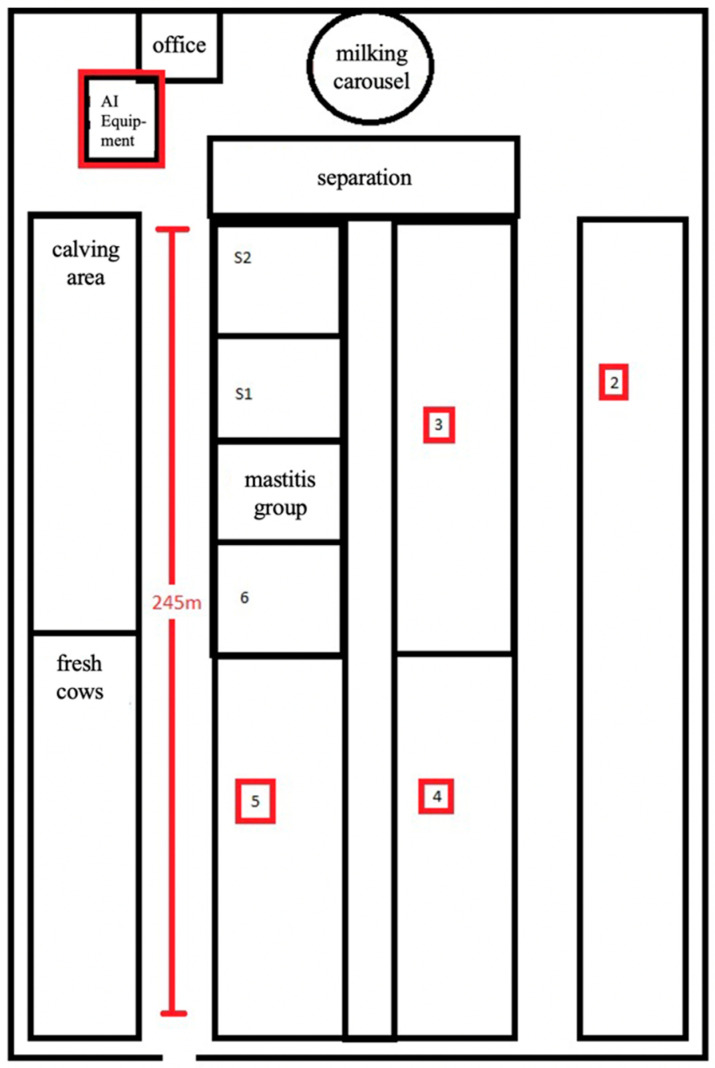
Schema of the experimental farm building. Areas relevant to the experiment are marked in red.

A total of 1159 cows in different lactations from one dairy farm in eastern Germany were included in the study. Over the period of 1 year, 3393 artificial inseminations were performed with frozen bull semen of 35 from 42 available sires. Both cows and bulls were of the Holstein-Friesian breed. The experimental animals were housed in a cubicle housing system with a total of approximately 1400 lactating cows (the difference between the total of 1468 lactating cows and the number of animals included in the investigation (*n* = 1159) resulted from the ineligibility of previously inseminated or early pregnant cows for participation), divided into six groups, according to their lactation and pregnancy status. Moreover, the operation was equipped with a 70-cow carousel, a selection area right next to the milking parlor, a calving area, and a separate room for the insemination equipment that was located between the milking parlor and the housing system. The entire length of the barn building measured 245 m, resulting in long distances for AI, even though most AI were performed in the separation area (Figure 1). 

The barn had large open side walls equipped with adjustable wind shutters. In addition, fans in the cubicle housing area and a sprinkler system in the waiting area were installed to reduce potential heat stress for cows under hot conditions. All dairy cows were milked 3 to 4 times a day and fed with identical total mixed ration containing maize, grass, straw, alfalfa, corn-cob-mix and shredded rye of in-house production [24]. 

### 2.2. Experimental Design

The investigation started in October 2017 and criterion for including dairy cows in the study was an absence of previous inseminations in the current lactation. Initially, the animals were divided into three groups of methods, each representing a different thawing regimen, according to their ear tag ID. The decimal number obtained when dividing the ear tag ID number by three, was set as the assignment criterion and cows with different lactation status were randomly distributed equally among these groups. Once categorized, the cows remained in the same group throughout the experiment, which ended when cows were either pregnant or culled. 

For heat detection Alta Cow Watch neck-tag sensors (Alta Deutschland GmbH, Uelzen, Germany) were employed, whereby estrus detection rate is at least 90% as confirmed by the manufacturer, and sensor data were verified by technicians based on heat symptoms. Automatic mating of cows to one or two sires was calculated using the Triple A system (Animal Analyses Associates), pairing each cow with a suitable bull based on their body traits to ensure well-balanced offspring. Beyond that, OvSynch (Application of GnRH at day 0, PGF_2α_ on day 7, GnRH at day 9, insemination after 18–20 h) was applied to cows that had not yet been inseminated by day 80 postpartum or were identified as non-pregnant 35 to 42 days after AI.

For our study, all AI were performed for reproduction purposes of the dairy herd, we additionally varied the used thawing method, which does not require any approval (Directive 2010/63/EU of the european parliament and of the council of 22 September 2010 on the protection of animals used for scientific purposes Article 3).

### 2.3. Semen Thawing

To assess the effect of different thawing methods on the fertility of frozen-thawed bull semen, AI was performed once a day in the morning with 0.25 mL straws from Alta Genetics Deutschland GmbH, a CSS certified breeding company, containing about 20 million sperm cells. Semen quality was evaluated by sperm morphology and motility before freezing and additional quality control was performed after defrosting, based on viability and motility of random samples of each batch.

Depending on the group, three different methods were applied:

Method A: Semen thawed in 38 °C warm water bath for 11 s; transport of semen portion in insemination gun and pre-warmed “gun warmer” (35 °C)

Method B: Semen thawed in 38 °C warm water bath for 35 s; transport of semen portion in insemination gun and pre-warmed “gun warmer” (35 °C)

Method C: Transport of frozen semen portion in insemination gun and non-warmed “gun warmer”; thawing of semen portion in the vagina or cervix of the cow for 30 s prior to application into the cows’ uterus.

A CITO thaw (CITO-Products, Inc., Watertown, WI, USA) was used to thaw the semen portions, constantly keeping the temperature-controlled water bath at 38 ± 0.2 °C. In addition, a tweezer, a timer, clean paper towels and a scissor were used for thawing procedure. Insemination guns were transported in an A.I. Gun Warmer (EM Tools, Inc., Rusk, TX, USA). For method A and B the gun warmer was heated, constantly keeping the inner temperature at 35 ± 1 °C. For method C the gun warmer was only used as a transportation pocket.

### 2.4. Insemination

Experimental cows were inseminated at least 50 days postpartum after the Alta Cow Watch alert and observation of standing heat and genital health by eight trained technicians of Alta Deutschland GmbH. Overall, 94% of the AI were performed by two main technicians. Via recto-vaginal method, the semen was deposited intrauterine closely to the cervix or intracervical, in the case of uncertain estrus symptoms, to avoid the risk of possible pregnancy loss from previous inseminations.

### 2.5. Data Collection

For every cow, pregnancy diagnosis was assessed by rectal palpation and transrectal ultrasonography with easi-scan ultrasound scanner and linear probe (128 elements; imv imaging, Phoenix crescent, Strathclyde Business Park, Bellshill, Scotland) 35–42 days after AI by the local veterinarian. Subsequently, the CR was calculated based on these results.

The herd management software Dairy Comp 305 (VAS, Tulare, CA, USA) was used for data collection: cow age, lactation number, insemination number, inseminator, sire, date, and days in milk (**DIM**) were recorded for each insemination. Moreover, ambient temperature data of the experimental days were obtained from the weather records of www.wetterkontor.de (accessed 17 January 2019).

### 2.6. Statistical Analyses

For statistical analyses, SAS Enterprise Guide 7.1, SAS, version 9.4 (SAS Institute, Inc., Cary, NC, USA), and Microsoft Excel for Office 365 were used. 

Both the cows in total and according to the group of different thawing methods were considered. To minimize outliers, only the first five AI per cow were analyzed. The number of animals with more than 5 AI was equally distributed among all three method groups. 

Normal distribution for calving-to-conception interval (**CCI**), days to first service (**DFS**), AI number, lactation number, and DIM at last AI were tested using the Shapiro–Wilk test. Since the data were not distributed normally, the median, as well as the 1st and 3rd quartiles, were calculated using descriptive statistics.

The Kruskal–Wallis test was used to evaluate a possible influence of the thawing method on CCI. In addition, chi-square test was applied to compare the proportions of cow status, number of AI, distribution of OvSynch and estrus AI, successful first AI according to thawing method, pregnancy loss, as well as the distribution of AI among the AI technicians and lactation numbers by thawing method, and for comparison of the insemination success according to the sires. Moreover, the Cochran’s trend test was used for the distribution of final cow status at the end of the experiment, sorted by the first five lactation numbers.

To evaluate the influence of several parameters on their success of individual AI, a binary generalized linear mixed model (**GLMM**) with an individual random intercept was used. In the first step, the main effects: thawing method, mean ambient temperature (at day of AI), AI technician, month of AI, number of lactations, DIM (at day of AI), number of AI, number of cycles (based on an assumed cycle duration of 21 days); and the interactions of these parameters with the thawing methodology, were considered. Parameters and interactions with a *p*-value of *p* < 0.15 in the first step, as well as the different thawing methods, were evaluated in the second step. *p*-values ≤ 0.05 were considered significant.

## 3. Results

Between October 2017 and December 2018, out of a total of 1468 cows, with average milk yield of 34.1 L per cow and day, 1159 fulfilled the requirements for study participation and were included in the investigation. The animals were distributed into three groups of methods as follows: group A: *n* = 426 (11 s, 38 °C water bath); group B: *n* = 348 (35 s, 38 °C water bath); group C: *n* = 385 (30 s, “in the cow”); corresponding to 3393 experimental AI. Due to culling, a pregnancy diagnosis (35–42 days after AI) was not determined in 56 AI. 

### 3.1. Descriptive Data

In the following, only the first five AI per cow (*n* = 3047) with pregnancy control (*n* = 2996) are described and evaluated. At the end of the study, 902 cows (77.8%) became pregnant, 179 cows (15.4%) remained open, and 78 animals (6.7%) left the experiment prematurely (died/sold).

### 3.2. Number of Lactations

The examined cows (*n* = 1159) completed a median of two lactations (1st quartile = 1.0/3rd quartile = 3.0). In addition, the proportions of the lactation groups were distributed homogeneously among the methods (*p* = 0.57).

### 3.3. Conception Rates

Out of 3047 AI, 902 (29.6%) resulted in pregnancy, 2094 (68.7%) were unsuccessful, and 51 (1.7%) could not be determined since the cows were culled before pregnancy check. According to the 2996 evaluable AI, these results provide an insemination success of 30.1%, with a median of 2 AI per cow (1st quartile = 1.0/3rd quartile = 4.0). 

**Figure 2:**
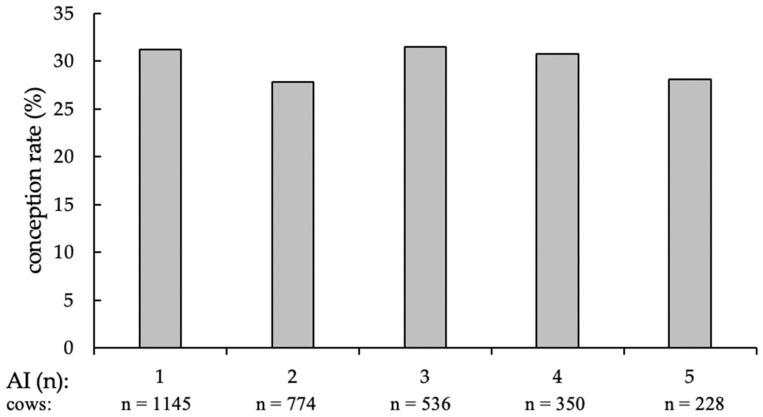
Conception rates of cows at first and additional AI (in %), separated by the number of AI and supplemented numbers of cows per group.

For the analyzable first AI (*n* = 1145; the number of first AI is lower than the number of animals included in the field trial (*n* = 1159), since 14 animals left the trial without pregnancy diagnosis), 357 resulted in pregnancy (CR = 31.2%); with the second AI (*n* = 774), 210 cows became pregnant (CR = 27.8%); the third AI (*n* = 536) produced 166 pregnancies (CR = 31.5%), the fourth (*n* = 350), 106 pregnancies (CR = 30.7%); and with the fifth and last AI (*n* = 228) 63 cows got pregnant (CR = 28.1%) (Figure 2).

**Figure 3:**
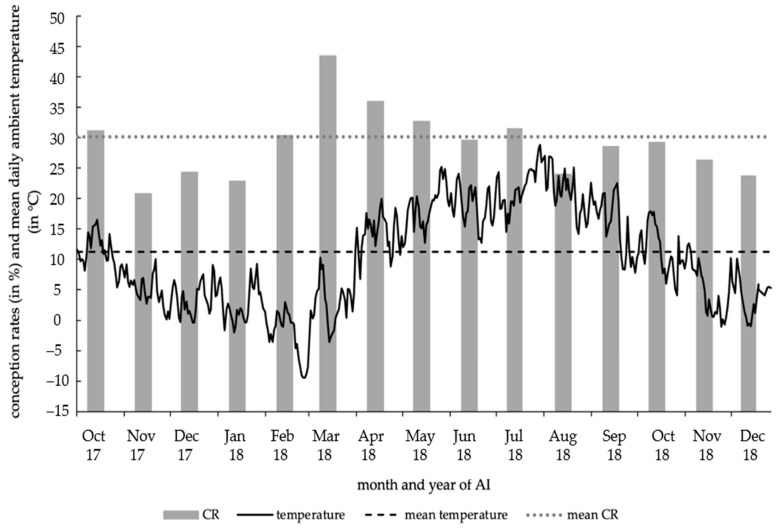
Conception rates of 2996 AI by month and daily ambient temperature (in °C).

The CR of experimental AI had its minimum in November 2017 with CR_min_ = 20.9% and maximized in March 2018 with CR_max_ = 43.6%. The ambient temperature of the individual days varied between a temperature minimum of −9.4 °C (28 February 2018–1 March 2018) and a temperature maximum of +28.8 °C (1 August 2018), averaging at +11.2 °C (Figure 3).

The distribution of all experimental AI throughout the study period, divided by months, is shown in Table 1.

**Figure 4:**
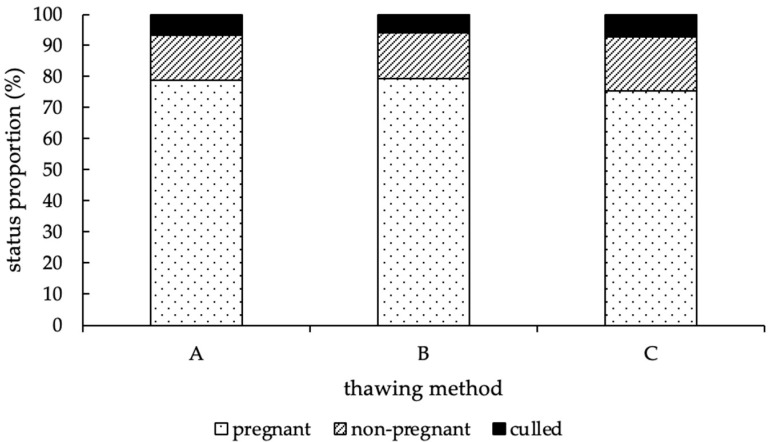
Distribution of the status proportions (in %) at the end of the study, according to the thawing method (A = 11 s, 38 °C water bath; B = 35 s, 38 °C water bath; C = 30 s, “in the cow”). No difference between method A, B, and C could be detected (*p* = 0.67).

At the end of the study, the status proportions of experimental cows were distributed equally among the different thawing methods (*p* = 0.67; Figure 4).

**Figure 5:**
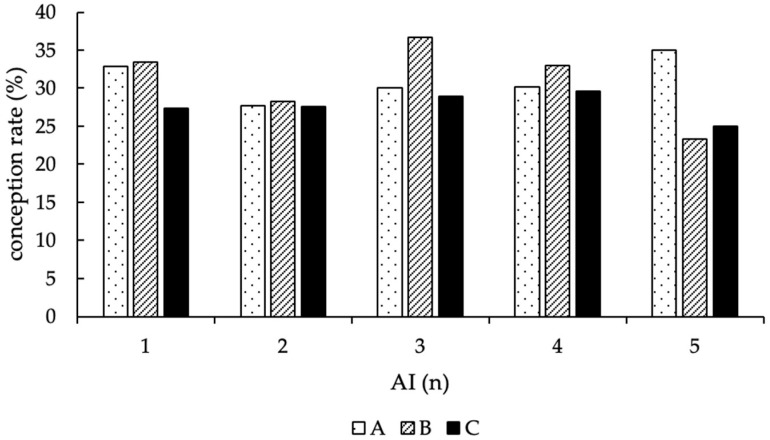
Distribution of the conception rates (in %) according to the number of AI and thawing methods (A = 11 s, 38 °C water bath; B = 35 s, 38 °C water bath; C = 30 s, “in the cow”).

Using thawing method A, 336 (78.8%) of the 426 animals became pregnant, 61 animals (14.3%) remained open, and 29 animals (6.8%) were sold or died during the experimental period. With thawing method B, 276 (79.3%) of 348 cows got pregnant, 51 (14.7%) remained open, and 21 (6.0%) left the farm before a pregnancy could be determined, and with thawing method C, 290 (75.3%) of 385 animals became pregnant, 67 (17.4%) remained open, and 28 animals (7.3%) were culled before pregnancy diagnosis (Figure 4). In total, group A contained 1107 AI, an average CR of 30.9%, and in median, two AI per cow (1st quartile = 1.0/3rd quartile = 4.0). Group B included 878 AI, an average CR of 31.9% and two AI per cow in median, as well (1st quartile = 1.0/3rd quartile = 4.0). In group C, 1062 AI were performed, thus resulting in an average CR of 27.8% and two AI per cow in median (1st quartile = 1.0/3rd quartile = 4.0). Moreover, no difference in fertility at first AI was detected among the thawing methods (*p* = 0.13). To compare the efficiency of the first and following AI, the distribution of the average CR, depending on the number of AI and thawing methods, is shown in Figure 5. 

With regard to the CR, depending on the different sires (with more than 30 performed AI), no differences (*p* = 0.53) could be determined based on the statistics.

### 3.4. Days to First Service and Calving-to-Conception Interval

In view of the DFS, no difference between group A, group B, and group C could be detected in the statistical analyses (*p* = 0.8; Table 2).

Regarding the average CCI of the cows, no statistically significant difference in the CCI of the several groups could be detected for the thawing methods, as well (*p* = 0.32; Table 2). 

### 3.5. Abortions

Among all animals included in the investigation, 121 out of 902 pregnant cows (13.4%) were aborted at an unknown time, so no distinction could be made between embryonic and fetal deaths. However, the comparison of pregnancy losses of the cows by thawing methods showed a highly significant difference between groups A and C (*p* = 0.007; Table 3).

### 3.6. Method of Artificial Insemination

**Figure 6:**
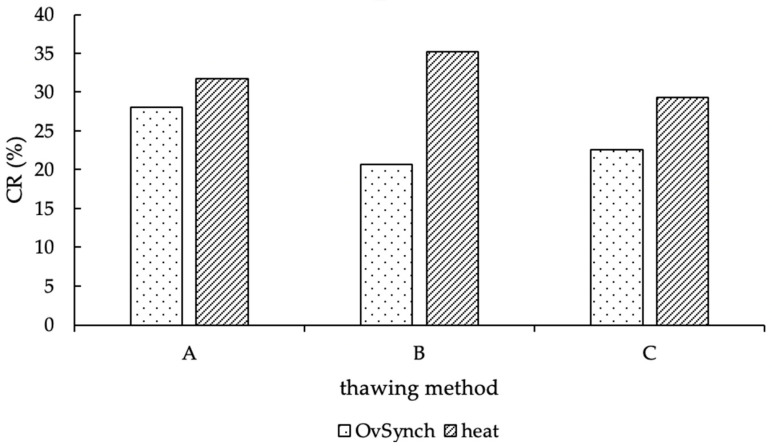
CR (in %) of natural heat and OvSynch-AI, according to the thawing methods (A = 11 s, 38 °C water bath; B = 35 s, 38 °C water bath; C = 30 s, “in the cow”).

Out of 2996, 691 AI (23.1%) were performed in a timed schedule, according to the OvSynch procedure; correspondingly, all other AI (*n* = 2302; 76.9%) were performed in natural heat (for 3 AI, no information was reported, whether cows were inseminated in natural estrus or during OvSynch program). In view of the proportion of estrus AI, 77.4% in method group A, 77.6% in group B, and 75.8% in method C were performed in natural heat. Consequently, 22.6% (group A), 22.4% (group B) and 24.2% (group C) were systematic breeding program inseminations. No difference between the proportions of these groups could be determined (*p* = 0.59). The CR of natural and systematic bred cows is shown in Figure 6, and the CR of OvSynch-AI is valued at 28.1% for method A, 20.7% for group B, and 22.6% for method C. In addition, the CR of natural heat AI was 31.8% in group A, 35.2% in group B, and 29.3% in group C (Figure 6).

### 3.7. Technicians

Regarding the AI technicians, only the ones that performed more than 50 AI during the entire experiment were considered in the statistical analysis (*n* = 4). According to that, the number of AI performed, and the CR achieved by each of them are shown in Table 4.

**Figure 7:**
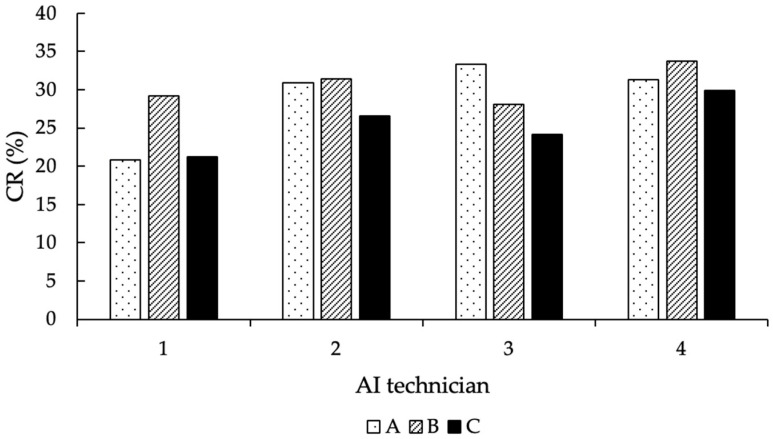
Distribution of the CR (in %) of the AI technicians, according to the thawing methods (A = 11 s, 38 °C water bath; B = 35 s, 38 °C water bath; C = 30 s, “in the cow”).

Overall, the number of AI performed by the four technicians was distributed equally among the thawing methods (*p* = 0.25), and their corresponding CR per thawing group is shown in Figure 7. 

### 3.8. Statistical Model and Interactions

The GLMM, with the inclusion of the random effect of the individuals, showed significant effects on the CR based on the performed AI method (natural heat AI vs. OvSynch-AI), the lactation number, and the month of insemination (Table 5 and Table 6). 

Beyond that, no other parameters showed significant effects on the CR, and no significant interactions could be determined (Table 5). Only one interaction, between the thawing method and the method of AI (natural heat AI vs. OvSynch-AI), showed a tendency to have a significant effect in the first step of the GLMM (*p* = 0.08; Table 5). 

**Figure 8:**
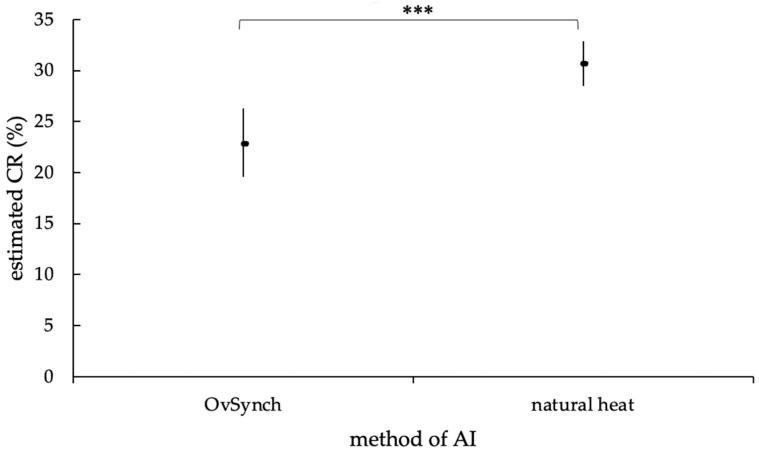
Estimated mean CR (in %) of OvSynch- and natural heat AI, with an upper and lower limit of the confidence interval, according to the statistic model with random effect. The estimated mean CR of the AI methods differed highly significantly (***, *p* = 0.0001).

The difference in the CR was highly significant depending on whether the AI was timed (OvSynch) or performed in natural estrus (*p* = 0.0001). Consequently, the chance for cows to become pregnant with a natural estrus AI was higher (mean (M) = 30.6%, lower limit of the confidence interval (lower bound, LB) = 28.5%, upper limit of the confidence interval (upper bound, UB) = 32.9%) than with a OvSynch-AI (M = 22.8%, LB = 19.6%, UB = 26.3%; Figure 8). 

Accordingly, the chance of a pregnancy due to an OvSynch-AI was 67% of the chance of a natural heat AI (odds ratio (OR) = 0.67). Furthermore, the lactation number of the animals had a highly significant influence on the CR (*p* = 0.0013); therefore, with increasing lactation number, the chance of becoming pregnant decreased (OR = 0.902).

**Figure 9:**
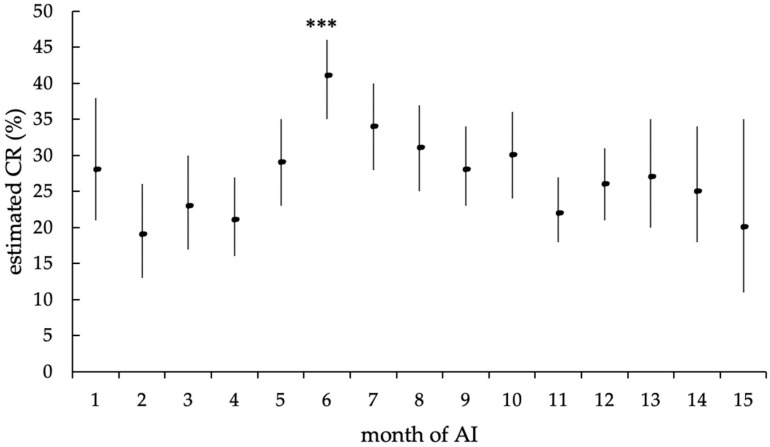
Estimated mean CR (in %) of the different months of AI, with upper and lower limits of the confidence interval, according to the statistical model with random effect. The estimated mean CR of the AI month (***) showed a highly significant difference from the estimated average CR (*p* < 0.0001).

Similarly, the month of the insemination showed a highly significant effect on the CR (*p* < 0.0001), as well. The calculated CR over the study period ranged from 19.0% in November 2018 to 40.6% in March 2018, thus, resulting in a significantly higher CR in March 2018 (*p* < 0.0001) compared to the estimated average CR ($\overset{-}{x}$ = 27.6%, Figure 9). However, mean ambient temperature did not affect conception rates (*p* = 0.67).

**Figure 10:**
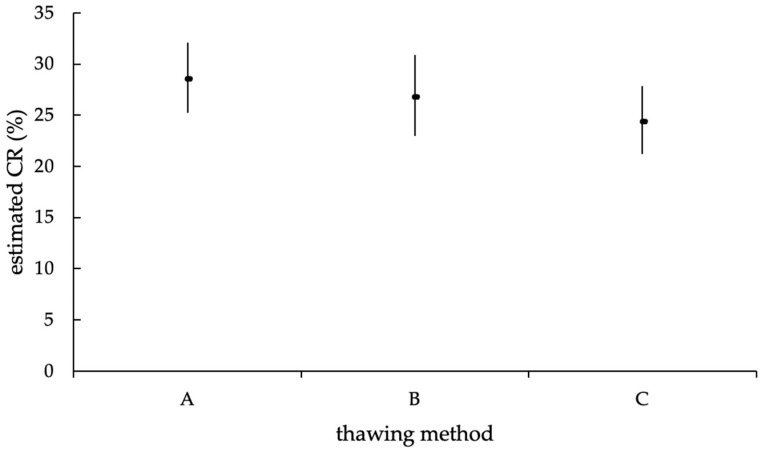
Estimated mean CR (in %) of the different thawing methods (A = 11 s, 38 °C water bath; B = 35 s, 38 °C water bath; C = 30 s, precervically/cervically), with upper and lower limits of the confidence interval, according to the statistic model with random effect. The estimated mean CR of the thawing methods was not statistically significantly different (*p* = 0.2).

Finally, considering the thawing method, no significant effect on the successfulness of AI could be found in the GLMM (*p* = 0.20), corresponding to a likelihood for the success of an AI of 28.5% with thawing method A (UB = 32.11%; LB = 25.20%), 26.7% for thawing method B (UB = 30.87%; LB = 22.98%) and 24.4% for method C (UB = 27.85%; LB = 21.22%; Figure 10).

## 4. Discussion

In recent decades, fertility issues have been identified as major problems in dairy herds, thereby resulting in being the main reason for culling dairy cows [25,26]. In general, the influencing factors for successful reproduction rates in the cattle herds can be divided into four main topic categories: (1) the count of animals detected in standing estrus for insemination, (2) inseminator efficiency, (3) fertility level of the herd, and (4) fertility level of the semen [27]. Nowadays, thawing methods for AI in dairy farms vary, and clear data to assess their influence on semen fertility are contradictory. Therefore, the aim of this study was to investigate the influence of three different thawing methods of frozen bull semen in AI of dairy cows on fertility under practical conditions and to determine further possible influencing factors on the success of AI.

### 4.1. Study Design

A large number of cows from one dairy farm were included in the study (*n* = 1159), wherefore their conditions differed only in group composition and milking time. Nevertheless, due to the distribution of the three thawing method groups, subdivided randomly by the ear tag number, no influence on the results by the milking time and the group composition is to be assumed. However, a possible limitation of the examined dairy farm might be the long distances between the inseminator room and the different cow groups due to the varying and partially increased time intervals. Despite this, the interval between semen thawing and performed AI was 15 min at maximum. In addition, possible further bias was minimized by equal distribution of lactation numbers between the thawing groups.

### 4.2. Conception Rate

To rate the success of AI, CR was used in the present study, although the true CR at the time of conception could not be determined based on the study methodology (pregnancy diagnosis at day 35–42 after AI). Previous investigations commonly used NRR for the evaluation of AI success, assuming that cows that do not return to service are generally pregnant. However, since this assumption is not accurate, CR is much more reliable for evaluating fertility. In some cases, cows are counted into the NRR, although reasons such as health restrictions, embryonic death, extended anovulatory periods, culling [28], or merely a poor estrus observation are the true cause for their absent heat [29]. Conversely, a low percentage of pregnant cows express signs of estrus, possibly further distorting the NRR [29]. A longer interval between AI and the calculation of the NRR implies increasingly similar outcomes compared to the true pregnancy rates; however, Busch and Waberski assumed in 2007 that there is about a 10% difference between the rate of successful inseminations and NRR [30]. Therefore, for a valid comparison of AI success between different thawing methods, it is more accurate to consider the CR as the target parameter with an additional evaluation of the culled animals. Concluding, the calculated average CR of the experimental animals corresponds to the value variance determined by another study of dairy farm fertility conducted in the same German region [31].

### 4.3. Seasonal Effect

An interesting outcome of the study is the highly significant effect of the month of AI on the CR. The statistical analyses revealed a higher CR in spring and early summer compared to late summer, autumn and winter. Seasonal influence on the CR of dairy cows is already described in the literature, and the possible reasons are various but still not completely identified [32,33]. In the present study, no effect of the mean daily ambient temperature could be shown, so the seasonal effect might be caused by a variety of management factors, feed quality, daily daylight length, weather, or the accumulation of health issues. On a closer examination of the study results, it is conspicuous that the highest CR was reached in the months with increasing daylight length. In the literature, the influence of daylight length on the fertility of dairy cows has been discussed previously, and it is known that daylight length has positive effects on estrus expression and fertility results in cattle [34,35]. 

### 4.4. Service Rate and Calving-to-Conception Interval

In addition, the statistical analyses showed relatively suitable service rates and CCI in the experimental farm, compared to the results of the dairy fertility study of Rethmeier, in 2019 [31]. According to the literature, improved estrus detection can lead to lower service rates and CCI, so the experimental farm’s heat detection system could be responsible for the improved parameters [36].

### 4.5. Abortions

Moreover, abortion occurred in 13.4% of the examined cows after a previous pregnancy diagnosis. Due to the study design, it could not be distinguished whether the pregnancy loss was caused by embryonic mortality (≤42 days after AI) or abortion (>42 days after AI). On the farm, a further pregnancy check was performed at day 70 after AI; however, for the statistical analyses, it was only recorded whether a pregnancy loss occurred, which could have also been the consequence of a further heat or a seen abortion. 

The highest risk for pregnancy loss in dairy cows is in the first four weeks after AI, whereby most abortions occur in the first week after conception [37,38]. Compared to research data from the USA and Spain, the present abortion rate is relatively high. Since the possible reasons for pregnancy loss in dairy cows are diverse and include both infectious and non-infectious risk factors, no decision on the causes could be made regarding the high abortion rate of the present study [39,40]. 

Interestingly, a significant difference in the proportions of pregnancy losses was found between thawing methods A and C (A = 17.6%, C = 9.0%; *p* = 0.007). The requirement for the thawing method to be the reason for increased abortion rate is sperm damage occurring in the period between thawing and AI, which would allow fertilization of the oocyte, but prevent permanent survival and successful development of the embryo. Laboratory studies focusing on different thawing methods for frozen semen could already demonstrate their damaging effect on spermatozoa [41,42]. Although their influence on fertility is partly controversial, it is conceivable but speculative that embryonic development may be affected by the thawing method [43,44]. 

Contradictory, several studies were able to determine fewer acrosome abnormalities, increased forward motility, and increased percent of motile spermatozoa during faster thawing in a water bath (corresponding to method A) compared to slower thawing (corresponding to method C), [41,45,46,47,48,49,50,51]. To verify a causality between the semen thawing method and the abortion rate, further studies will be necessary.

### 4.6. Method of Artificial Insemination

Contrary to existing research data, the results of the present study showed significantly higher CR in cows inseminated in natural estrus compared to timed AI [52]. According to the literature, the OvSynch procedure is particularly advantageous in farms, where suitable estrus observation cannot be guaranteed, as it can increase the PR with generally performed OvSynch [53]. In Europe, the general use of hormones in livestock production is socially unaccepted, wherefore the OvSynch program is mostly used for cows, not showing natural estrus symptoms. As noted before, the missing expression of a natural heat can be a consequence of manifold reasons, such as fertility issues or general health problems, which could also be the explanation for a lower CR in the examined cows that underwent the OvSynch program. 

Additionally, although not significant, the statistical analyses showed a tendency for interaction between the thawing method and the insemination method in the first step of the GLMM. According to that, the tendency for interaction shows that the AI success of method A tends to be less dependent on the AI method compared to methods B and C. However, this tendency was no longer detectable in the second step of the model, as other influencing factors have a more decisive effect on the conception rate. Nevertheless, it is conceivable that there might be a significant interaction between the thawing method A and OvSynch-AI, although the reason is currently intangible for the authors. Further research is needed to answer this question in the future.

### 4.7. Thawing Method

The main focus of the study was to analyze the influence of the different thawing methods in AI of dairy cows on CR under practical conditions. Therefore, inaccuracies in timing, different distances and intervals during AI, and different durations of work processes between the various technicians could only be recorded and evaluated to a limited extent. Nonetheless, the conditions, in this respect, were the same for all three methods.

At the end of the investigation, the status proportions (pregnant, open, and culled cows) were homogenously distributed among the different thawing methods (*p* = 0.67). Furthermore, no significant effect of the different thawing methods on the CR could be detected. These results are consistent with other studies that could not detect any differences or merely tendencies between short and long water bath thawing of frozen bull semen on the NRR or between thawing in a water bath and “in the cow” on the CR [23,54,55]. In contrast, several investigations detected better AI success when semen was thawed for a longer time in a warm water bath, compared to shorter thawing, on the 75-day or 66-day NRR [13,14]. Comparatively, the amount of AI in these studies was much larger than the sample size used in the present study. It is known that the likelihood of a result becoming significant increases with a growing sample size [56]. Nevertheless, not every result of the thawing method experiments showed significant effects, despite the large sample size. Furthermore, it should be noted that the previously mentioned studies used different statistical models and that their results were influenced by a wide variety of statistical adjustments, wherefore a direct comparison of the different study results is not possible. Accordingly, in most field trials, the fertility of differently thawed semen was evaluated using the NRR, which, as explained earlier, is less suitable than the evaluation by the CR [13,14,15,16]. 

## 5. Strengths and Limitations

However, it is important to say that a possible limitation of the present study may have resulted from the use of a pre-warmed gun warmer. Consequently, possible differences between thawing methods A and B may have been attenuated due to the time spent in the gun warmer and the equalization of the semen temperature. 

Moreover, sperm samples were not analyzed according to different thawing methods, and no data on semen quality during sperm analysis before and after freezing were collected to confirm sperm quality. 

A further limitation of the study is that the semen of different bulls was used. The use of a single bull with identical semen quality would have been desirable, but in dairy practice, this is usually not possible over the period of one year due to farmers’ preferences.

## 6. Conclusions

Under present practical conditions, no differences between three different thawing methods could be detected, so no clear recommendation for semen thawing method in dairy reproduction can be made based on our data. 

Concluding, inadequate reproductive performance of high-producing, lactating dairy cows is a multifactorial consequence of farm and fertility management, wherefore farmers, as well as breeding companies, should focus more on important fertility influencing factors related to the cow, e.g., increasing percentage of cows showing natural heat to improve the reproduction performance of dairy cows in the future.

## Figures and Tables

**Table 1:** Number and proportion of AI per month and year during the study.

Month/Year of AI	Number of AI	Proportion of AI (%)
Oct. 17	109	3.6
Nov. 17	134	4.5
Dec. 17	164	5.5
Jan. 18	205	6.9
Feb. 18	230	7.7
Mar. 18	303	10.1
Apr. 18	247	8.2
May 18	244	8.1
Jun. 18	229	7.6
Jul. 18	228	7.6
Aug. 18	283	9.5
Sep. 18	311	10.4
Oct. 18	157	5.2
Nov. 18	110	3.7
Dec. 18	42	1.4
**∑**	2996	100

**Table 2:** Median, 1st, and 3rd quartile (in days) of DFS and CCI, according to all cows, group A, B, and C.

Effect	1st Quartile (D)	Median (D)	3rd Quartile (D)
Average DFS of all cows	58	67	85
DFS group A	58	68	84
DFS group B	58.5	66	84.5
DFS group C	58	67	86
Average CCI of all cows	69	93	128
CCI group A	70	92	128.5
CCI group B	66.5	93	124
CCI group C	71	95	132

**Table 3:** Total of pregnant cows, number of pregnancy losses, and pregnancy loss rate (in %), according to the thawing method (A = 11 s, 38 °C water bath; B = 35 s, 38 °C water bath; C = 30 s, “in the cow”).

Thawing Method	Pregnant Cows	Pregnancy Loss	Rate of Pregnancy Loss (%)
A	336	59	17.6 ^a^
B	276	36	13.0 ^ab^
C	290	26	9.0 ^b^

Numbers with different superscripts are statistically significantly different (*p* < 0.01).

**Table 4:** Conception rates (in %) and number of AI performed by the different AI technicians.

**AI Technician**	**CR (%)**	**Total**
1	23.5	81
2	29.6	1595
3	28.6	91
4	31.5	1205

**Table 5:** Results of the first step of the GLMM with the inclusion of the random effect.

Effect	DF	F	P
Thawing method	2	0.25	0.78
Month of AI	14	3.25	<0.0001
Method of AI (natural heat/OvSynch)	1	12.47	0.0004
Number of lactation	1	10.40	0.0013
DIM	1	0.00	0.97
Number of AI	4	0.66	0.62
Cycle	1	0.02	0.88
Mean ambient temperature	1	0.18	0.67
AI technician	3	0.51	0.68
Method*method of AI	2	2.53	0.08
Method*month of AI	28	0.98	0.50
Method*number of AI	8	0.81	0.59
Method*DIM	2	0.11	0.89
Method*mean ambient temperature	2	0.32	0.73
Method*cycle	2	0.13	0.88
Method*number of lactation	2	0.13	0.88

**Table 6:** Results of the second step of the GLMM with the inclusion of the random effect.

Effect	DF	F	P
Thawing method	2	1.59	0.20
Method of AI (natural heat/OvSynch)	1	15.06	0.0001
Month of AI	14	3.45	<0.0001
Number of lactation	1	10.43	0.0013
Thawing method*method of AI	2	2.03	0.13

## Data Availability

The data presented in this study are available on request from the corresponding author. The data are not publicly available due to privacy restrictions of the participant.
